# Perspective on Pediatric/Congenital Cardiac Catheterization in Response to the First Wave of COVID∗

**DOI:** 10.1016/j.jacadv.2022.100150

**Published:** 2022-12-30

**Authors:** Holly Bauser-Heaton

**Affiliations:** Children’s Healthcare of Atlanta Cardiology, Department of Pediatrics, Atlanta, Georgia, USA

**Keywords:** congenital interventional cardiology, COVID

**Figure:**
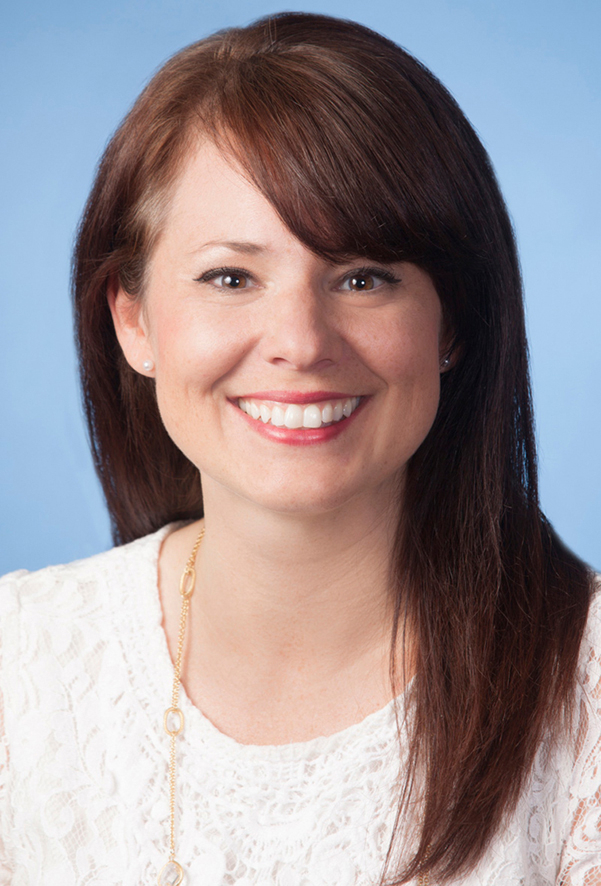

In the paper by Quinn et al^1^ in this issue of *JACC: Advances*, the impact of the first wave of COVID on outcomes in the congenital cardiac catheterization is explored. The authors nicely used an existing network, Congenital Cardiac Catheterization Project on Outcomes, to investigate the policy changes, case volume, complexity, and outcomes during the unexpected wave of COVID in its first couple months. Most centers around the world found this period to be particularly challenging with limitations in hospital-based resources and supply chain limitations. While there was no standard approach to this first wave of COVID, many congenital cardiac catheterization laboratories responded by performing only medically urgent cases, thereby increasing case complexity and decreasing overall volume. This approach primarily led to pre-reviewed, higher-risk procedures being performed by a core group of catheterization laboratory staff and physicians. While many have appreciated the increased stress system-based issues created through the demand of performing only high-risk procedures, the outcomes of these procedures had previously not been analyzed. This article uniquely compares procedural outcomes during the pre-COVID, first wave of COVID, and post-first-wave periods. Findings of stable to even improved overall outcomes despite the case-mix being unbalanced to high risk and reduced volumes were surprising.

Certainly, these findings naturally beg to ask the question, “Can we utilize these strategies to improve outcomes in the congenital cardiac catheterization laboratory?” The answer to this question may prove much more difficult than one might initially believe. Several confounders to this observed outcome improvement exist, including reduced overall case volume. As the core group of performing interventionalists remained the same in each period examined, the requirement for prereview and discussion of necessity was heightened during the first wave of COVID. Resource allocation undoubtedly varied among institutions, but nationally, the need to prioritize emergent procedures and largely pause on elective cases during the first wave was necessary in both congenital and adult catheterization laboratories.^2^ Prereview with attention paid to acuity and risk was required to allow resource allocation throughout hospital systems of varying degree. This act alone likely had a significant impact. As case volume increases and tasks per individual involved increase, the likelihood of knowing the case complexity more superficially also increases. During high-risk procedures, reduction of risk can be seen with intimacy of details to prevent and rescue from poor outcomes. The focus and attention paid to 1 case compared to several more likely proves valuable as well although this requires additional investigation. Interestingly, these findings have not been shown in adult interventional laboratories where, during the COVID-19 pandemic period, mortality was noted to increase for individuals who underwent percutaneous interventional procedures.^3^ One must admit, however, the general burden COVID placed on adult facilities was much higher than that on pediatric centers, and the viral effect on patient comorbidities cannot be discounted.

As we enter into a “long” COVID period, supply chain issues persist, particularly in the cardiac catheterization laboratory. Scarcity in resources result in the use of less-than-ideal materials and may result in higher complications as we move forward. During the first wave of COVID, we experienced shortage of materials used by all in the health care profession, such as gloves, masks, and gowns. However, it took some time into the post-first-wave period for the materials utilized for specialized care to become scarce. Balloons, wires, catheters, and even contrast have proven to be in limited supply, resulting in our field, in particular, utilizing supplies in an even more creative fashion. Already, the congenital space must use materials in an off-label fashion, but the current era of supply chain concerns increases our need for creativity, utilizing materials not intended for that particular use. While we have for decades been successful in this task of resourcefulness, this does often result in less-than-desirable outcomes if an item is too long (ie, length of balloon for angioplasty) or too large (ie, need for larger access for intervention in small patients) and can increase complication rates. The full effect of this is yet to be seen.

In addition, we are now facing staffing shortages throughout the country.^4^ Nursing and technician support has resulted in high turnover, fewer experienced staff, and the need for longer periods of training for support teams. This fact alone may lead to increased risk in all case types, let alone high-risk procedures. One of the benefits of the first wave of the COVID period may have been the centralized preprocedural reviews; however, with increased staff turnover, this experienced review may not be possible. Existing staff would be burdened with onboarding, training, education, and procedural review with a period of increased case volume compared to the first-wave COVID period.

While I do applaud the authors for this work and noting the improved outcomes during a unique period in our history, I question the applicability of these findings going forward. The odd nature of a nationwide shut down, limitations to emergency procedures, and, in some cases, severe limitation of the number of cases performed may not again happen in a lifetime. Therefore, the ability to apply this particular set of circumstances going forward may not be possible. The understanding that focus, time spent on individual procedures, skilled and cohorted staff, as well as intense preprocedural review should be valued and may lead to improved outcomes is one that should be taken into consideration in invasive cardiology.

## Funding support and author disclosures

Dr Bauser-Heaton is a proctor for W.S. Gore.
